# Bacterial amyloid curli acts as a carrier for DNA to elicit an autoimmune response via TLR2 and TLR9

**DOI:** 10.1371/journal.ppat.1006315

**Published:** 2017-04-14

**Authors:** Sarah A. Tursi, Ernest Y. Lee, Nicole J. Medeiros, Michael H. Lee, Lauren K. Nicastro, Bettina Buttaro, Stefania Gallucci, Ronald Paul Wilson, Gerard C. L. Wong, Çagla Tükel

**Affiliations:** 1 Department of Microbiology and Immunology, Lewis Katz School of Medicine, Temple University, Philadelphia, Pennsylvania, United States of America; 2 Department of Bioengineering, California Nano Systems Institute, University of California, Los Angeles, California, United States of America; 3 Department of Chemistry and Biochemistry, California Nano Systems Institute, University of California, Los Angeles, California, United States of America; McMaster University, CANADA

## Abstract

Bacterial biofilms are associated with numerous human infections. The predominant protein expressed in enteric biofilms is the amyloid curli, which forms highly immunogenic complexes with DNA. Infection with curli-expressing bacteria or systemic exposure to purified curli-DNA complexes triggers autoimmunity via the generation of type I interferons (IFNs) and anti-double-stranded DNA antibodies. Here, we show that DNA complexed with amyloid curli powerfully stimulates Toll-like receptor 9 (TLR9) through a two-step mechanism. First, the cross beta-sheet structure of curli is bound by cell-surface Toll-like receptor 2 (TLR2), enabling internalization of the complex into endosomes. After internalization, the curli-DNA immune complex binds strongly to endosomal TLR9, inducing production of type I IFNs. Analysis of wild-type and TLR2-deficient macrophages showed that TLR2 is the major receptor that drives the internalization of curli-DNA complexes. Suppression of TLR2 internalization via endocytosis inhibitors led to a significant decrease in *Ifnβ* expression. Confocal microscopy analysis confirmed that the TLR2-bound curli was required for shuttling of DNA to endosomal TLR9. Structural analysis using small-angle X-ray scattering revealed that incorporation of DNA into curli fibrils resulted in the formation of ordered curli-DNA immune complexes. Curli organizes parallel, double-stranded DNA rods at an inter-DNA spacing that matches up well with the steric size of TLR9. We also found that production of anti-double-stranded DNA autoantibodies in response to curli-DNA was attenuated in TLR2- and TLR9-deficient mice and in mice deficient in both TLR2 and TLR9 compared to wild-type mice, suggesting that both innate immune receptors are critical for shaping the autoimmune adaptive immune response. We also detected significantly lower levels of interferon-stimulated gene expression in response to purified curli-DNA in TLR2 and TLR9 deficient mice compared to wild-type mice, confirming that TLR2 and TLR9 are required for the induction of type I IFNs. Finally, we showed that curli-DNA complexes, but not cellulose, were responsible elicitation of the immune responses to bacterial biofilms. This study defines the series of events that lead to the severe pro-autoimmune effects of amyloid-expressing bacteria and suggest a mechanism by which amyloid curli acts as a carrier to break immune tolerance to DNA, leading to the activation of TLR9, production of type I IFNs, and subsequent production of autoantibodies.

## Author summary

Bacterial amyloids are conserved proteins expressed by many bacteria in biofilms. Bacterial amyloid curli and DNA form highly immunogenic complexes that stimulate autoimmunity and accelerate the progression of systemic lupus erythematosus. Here, we show that the innate immune receptors TLR2 and TLR9 are critical for shaping the autoimmune adaptive immune response to curli-DNA complexes. Mice deficient in these receptors show attenuated production of anti-double-stranded DNA autoantibodies and type I IFNs. The cross beta-sheet structure of curli is recognized by TLR2, leading to endosomal internalization of the curli-DNA complex and subsequent binding to TLR9. Synchrotron diffraction studies suggest that curli-DNA immune complexes present double-stranded DNA rods at an inter-DNA spacing that matches well to the steric size of TLR9, thus promote multivalent amplification of binding and TLR9 activation. Overall, our results identify a novel series of events pivotal to induction of autoimmunity by amyloid-expressing bacteria.

## Introduction

Amyloid proteins, such as human amyloid beta and serum amyloid A, self-assemble into a cross-beta sheet quaternary structure, in which the individual strands of the beta sheets are oriented perpendicularly to the fiber axis [1, 2]. Like humans, bacteria also produce amyloids. It is estimated that over 40% of bacterial species produce amyloids, and these proteins are major structural components of biofilms [3] [4]. Biofilms are defined as communities of bacteria encapsulated in a self-produced extracellular matrix [5]. Biofilms can form during infection and can be difficult to eradicate [6–9].

Originally described in the 1980s, curli is one of the most well-studied bacterial amyloids; curli is expressed by members of the Enterobacteriaceae family such as *Salmonella enterica* serovar Typhimurium and *Escherichia coli* [10]. Research has shown that without the expression of curli, due to deletions in the *csgA* gene (which encodes the major subunit of curli), enteric biofilms are defective [11]. The biogenesis of curli is regulated through two bidirectional operons: *csgDEFG*, which encodes a regulatory protein as well as proteins that aids in the assembly of curli, and *csgBAC*, which codes for the major structural proteins CsgA and CsgB [4, 12]. CsgA and CsgB are co-secreted across the plasma membrane. First, CsgB nucleates and attaches CsgA to the cell surface. Then, soluble unpolymerized monomeric CsgA polymerizes with the cell surface bound CsgA, forming the core of the amyloid beta sheet secondary structure [13–15].

We have previously shown that the fibrillar amyloid structure of curli is recognized by Toll-like receptor 2 (TLR2) [16–18]. TLRs are pattern recognition receptors (PRRs) expressed by innate immune cells, including dendritic cells and macrophages, that detect conserved pathogen associated molecular patterns (PAMPs). TLRs reside on both the cell surface (TLR2, TLR5, and TLR4) and within intracellular compartments (TLR3, TLR7, and TLR9). Activation of TLRs leads to the transcription of *NF-κB* regulated genes and interferon-regulated genes, leading to the initiation of the innate immune response [19]. The TLR2/1/CD14 heterocomplex recognizes the beta sheet secondary structure of curli and activates *NF-κB*, eliciting the production of proinflammatory chemokines and cytokines including IL-8, IL-6, and IL-17A [16–18, 20, 21]. In addition to the TLR2/1/CD14 heterocomplex, curli is also recognized by the NLRP3 inflammasome, which leads to the activation of caspase-1/11 and the maturation of pro-IL-1β to IL-1β [22]. Recently, our group showed that biofilms of *S*. Typhimurium not only contain the amyloid curli but also contain extracellular DNA bound to curli in the form of insoluble, ultra-stable curli-extracellular DNA complexes; the DNA promotes bacterial amyloid formation by increasing the fibrillization rate of synthetic curli monomers into mature fibrils [23]. Recently, we demonstrated that curli-DNA complexes found in enteric bacterial biofilms accelerate the progression of autoimmunity in a murine model of a human autoimmune disease, systemic lupus erythematosus (SLE), as these complexes induce the generation of autoantibodies and a type I interferon (IFN) response [23].

TLR9 is known to recognize bacterial DNA through binding to unmethylated cytosine-guanine (CpG) dinucleotides [24]. The activation of TLR9 leads to the production of type I IFNs, a family of cytokines important for both bacterial and viral infections that trigger pleiotropic activation of the immune system [25]. Type I IFNs have been shown to be pathogenic in SLE. Peripheral blood mononuclear cells (PBMCs) from SLE patients overexpress type I IFNs and ISGs in what is referred to as the “IFN signature” [26]. In murine models of lupus, the administration of IFNα accelerates lupus onset [27], whereas deletion of the gene encoding the type I IFN receptor (IFNAR) leads to a milder, delayed disease [28]. The induction of the IFN signature could be the result of an inheritable disease risk factor [29], and it could also be amplified by the autoimmune activation of endosomal TLR7 and TLR9 by immune complexes containing lupus-associated autoantibodies and self-nucleic acids [30–32].

Here, using a multidisciplinary approach, we show that immune activation by curli-DNA complexes is a two-step process requiring engagement of the innate immune receptors TLR2 and TLR9. First, TLR2 mediates internalization of the curli-DNA complex into the endosomes of immune cells. In the second step, the structure of the curli-DNA complex enables multivalent presentation of parallel double-stranded DNA (dsDNA) rods to endosomal TLR9, leading to amplification of binding, production of type I IFNs, and induction of autoantibodies.

## Results

### Type I interferon response elicited by curli-DNA complex is dependent on TLR2 and TLR9

**Fig 1:**
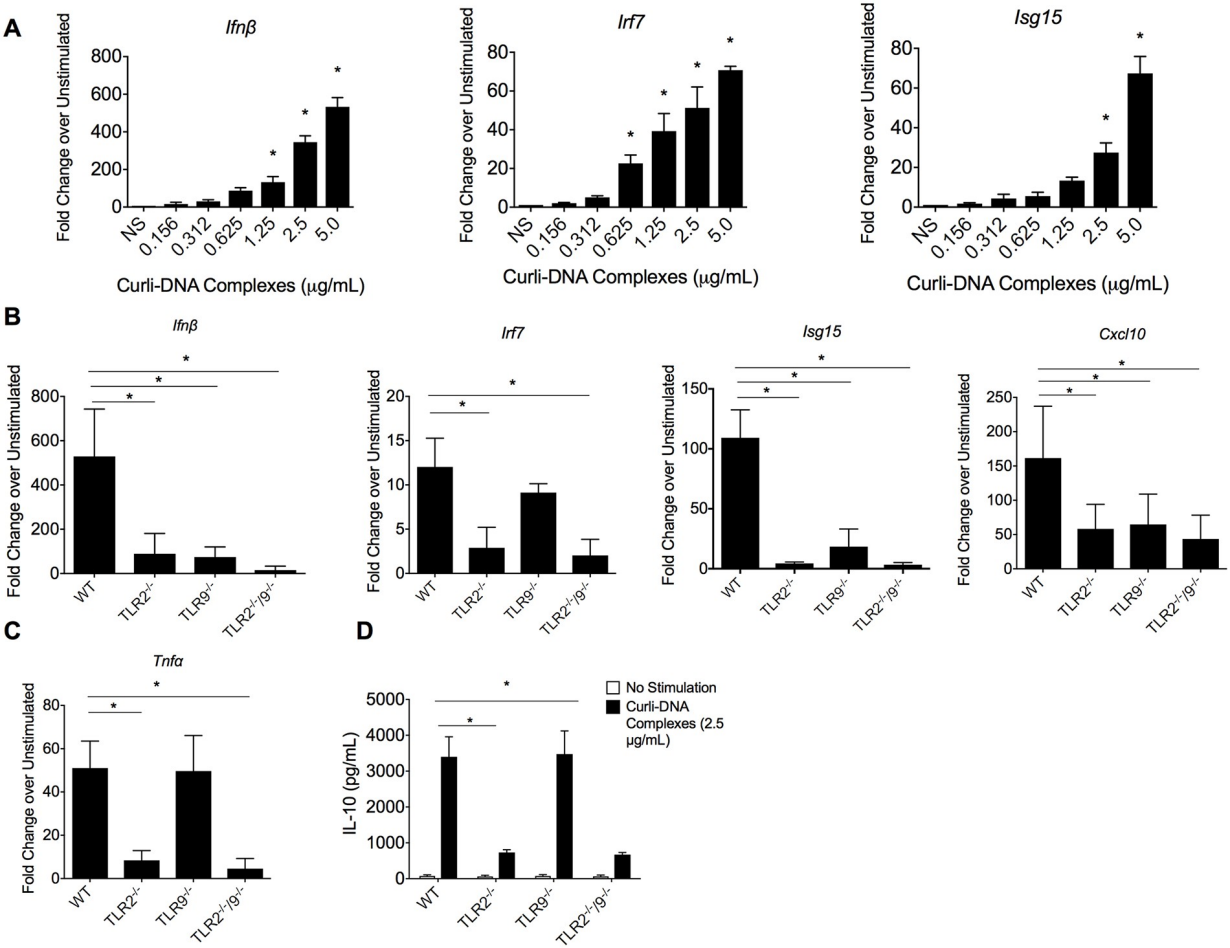
Curli-DNA complexes elicit a type I interferon response in innate immune cells in a TLR2/TLR9 dependent fashion. **A**. Wild-type IMMs (1x10^5^ cells) were stimulated with increasing concentrations of curli-DNA complexes purified from *S*. Typhimurium IR715 *msbB* (0, 0.5, 1.0, 1.5, and 2.5 μg/mL) for 3 hours, and levels of *Ifnβ (*left panel), *Irf7* (middle panel), and *Isg15* (right panel) were determined by qPCR. Wild-type (WT), TLR2^-/-^, TLR9^-/-^, and TLR2^-/-^/TLR9^-/-^ IMMs (1x10^5^ cells) were stimulated with 2.5 μg/ml of curli-DNA complexes for 3 hours. (**B**) *Ifnβ*, *Isg15*, *Irf7*, and *Cxcl10* levels were determined by qPCR. (**C**) *Tnfα* mRNA levels were determined by qPCR. **D**. IL-10 production was quantified by ELISA in the supernatants. Mean and SE were calculated from results from at least three independent experiments. * p <0.05 as determined by Students t-test.

The extracellular matrix of *S*. Typhimurium biofilm is composed mainly of curli fibers, cellulose, and extracellular DNA [23, 33]. Recently, we showed that curli and DNA form complexes in biofilms and that purified curli-DNA complexes stimulate the type I IFN responses in dendritic cells [23]. Macrophages, another important myeloid population, produce high amounts of type I IFNs, IFNα and IFNβ. To evaluate the effect of curli-DNA complexes on macrophages, we first stimulated wild-type murine immortalized macrophages (IMMs) with increasing concentrations of curli-DNA complexes for 3 hours, a time point after stimulation at which macrophages express type I IFN related genes [34]. As the concentration of purified curli-DNA was increased, the levels of *Ifnβ* mRNA as well as mRNAs encoding other ISGs, *Irf7*, and *Isg15*, were increased (Fig 1A).

Although the purification method used here included multiple rounds of DNase treatment, a significant amount of DNA (300–500 ng per 1 mg protein) was still found associated with curli fibers (S1 Fig). When we tested a secondary purification method, sequential differential centrifugation [35], we still found that DNA associated with curli fibers confirming that this phenomenon was not due to the purification protocol (S1 Fig). The curli-DNA complexes purified by either the traditional or the sequential centrifugation method both generated similar levels of *Isg15 and Ifnβ* expression on IMMs (S1 Fig). These data indicate that curli-DNA complexes elicit the expression of type I IFN responsive genes in a dose-dependent manner in macrophages, one of the main antigen-presenting cell types.

TLR9 recognizes bacterial CpG motifs leading to the phosphorylation and translocation of transcription factors IRF3 and IRF7 in a MyD88-dependent manner [36]. Activation of TLR9 by bacterial DNA leads to the generation of type I IFNs [37]. To investigate if TLR9 is involved in the type I IFN response generated in response to DNA complexed within curli fibers, we stimulated wild-type and TLR9-deficient IMMs with 2.5 μg/mL of curli-DNA complexes purified from *S*. Typhimurium *msbB* biofilm, a dose that we have found to be a potent activator of the IFN response (Fig 1A). This *S*. Typhimurium mutant expresses a modified LPS that does not function as an effective TLR4 agonist [17]. Since the conserved beta sheet structure of curli activates the TLR2 [16–18, 23], we also assayed TLR2- and TLR2-9-deficient IMMs. After stimulating the IMMs for 3 hours, we determined levels of various ISGs as well as *Tnfα* by qPCR. Significant decreases in the transcript levels of *Ifnβ*, *Irf7*, *Isg15*, and *Cxcl10* were detected in TLR2^-/-^, TLR9^-/-^, and TLR2^-/-^/TLR9^-/-^ macrophages (Fig 1B), suggesting that both TLR2 and TLR9 are important for recognition of curli-DNA complexes.

To test whether curli activates TLR2, we determined the transcript levels of NF-κB-dependent *Tnfα*, as well as the protein levels of IL-10 in the supernatant of wild-type, TLR2^-/-^, TLR9^-/-^, and TLR2^-/-^/TLR9^-/-^ IMMs stimulated with 2.5 μg/mL purified curli-DNA complex. The transcript levels of *Tnfα* were lower in the TLR2^-/-^ and TLR2^-/-^/TLR9^-/-^ stimulated macrophages than in stimulated wild-type IMMs, whereas levels of *Tnfα* were similar after stimulation in TLR9^-/-^ and wild-type IMMs (Fig 1C). We found significantly lower levels of IL-10 in the supernatants of TLR2^-/-^ and TLR2^-/-^/TLR9^-/-^ stimulated macrophages compared to stimulated wild-type and TLR9^-/-^ macrophages (Fig 1D). Similar data was obtained using bone marrow-derived macrophages (S2 Fig). Overall, these data suggest that both TLR2 and TLR9 are activated by curli-DNA complexes and that both receptors are involved in the generation of type I IFNs. As shown previously [38], TLR9 was not required for the early expression of NF-κB-dependent cytokines.

### Curli-DNA complexes gain access to endosomal TLR9 via TLR2 engagement

**Fig 2:**
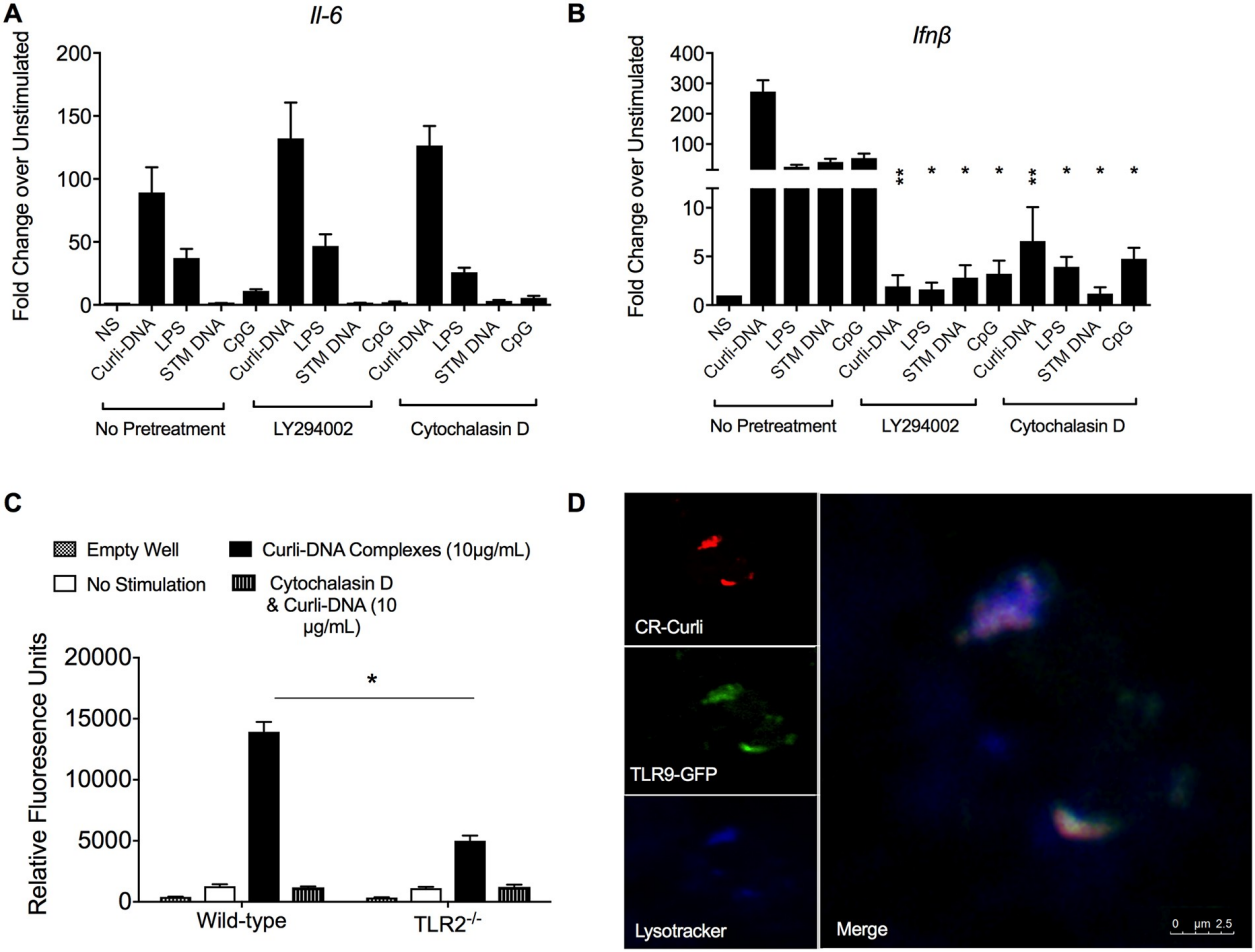
TLR2 serves as a carrier to bring curli into the cell to allow for endosomal TLR9 signaling. Wild-type IMMs (1x10^5^ cells) were pretreated for 1 hour with either 2.5 μM cytochalasin D or 50 μM LY294002 and then stimulated for 3 hours with, 2.5 μg/mL curli-DNA complexes (curli), 50 ng/mL LPS, 3.0 μg/mL STM genomic DNA, or 3.0 μg/mL CpG or were left unstimulated (NS). After 3 hours, cells were collected. (**A**) Levels of *Il-6* were determined by qPCR. (**B**) Levels of *Ifnβ* were determined by qPCR. **C**. Wild-type or TLR2^-/-^ IMMs (1x10^5^ cells) were pretreated with 2.5 μM cytochalasin D for 1 hour (or not) and then stimulated with 10 μg/ml of Congo red-labeled curli-DNA complexes for 1 hour. Cells were washed 3 times with sterile PBS and then lysed with 1% Triton-X in PBS and transferred to a 96-well black optical plate. Fluorescence was measured after excitation at 497 nm with emission at 614 nm. **D**. TLR9-GFP-expressing macrophages (1x10^6^ cells) were stimulated with 10 μg/mL of Congo red-labeled curli-DNA complexes and stained with 75 nM LysoTracker Blue for 10 minutes and then visualized using sequential scanning confocal microscopy (100x, oil immersion). LysoTracker Blue DND-22 was visualized at an excitation wavelength of 373 nm and emission of 422 nm, and Congo red-labeled curli was visualized at an excitation wavelength of 514 nm and emission of 650–750 nm. Scale bar represents 2.5 μM. Mean and SE were calculated by averaging results from three independent experiments. *p <0.05 as determined by Students t-test.

TLR9 resides in the endosomal compartment [39]. In order for curli-bound DNA to activate TLR9, the curli-DNA complex must enter the cell. Upon activation of TLR2, the receptor is internalized and localized into the endosome [40, 41]. We hypothesized that TLR2-bound curli brings the curli-DNA complex into the endosome where the DNA component activates TLR9. To test this hypothesis, we inhibited endocytosis to prevent internalization of TLR2 with two chemicals, cytochalasin D [42] and LY294002 [43, 44]. We pretreated wild-type IMMs with 2.5 μM cytochalasin D or 50 μM LY294002 for 1 hour or left cells untreated. IMMs were then stimulated with purified curli (2.5 μg/mL), LPS (50 ng/mL), CpG oligonucleotides (3.0 μg/mL), or *Salmonella* (STM) genomic DNA (3.0 μg/mL) for three hours. There was a significant upregulation of *Il-6 and Ifnβ* expression in cells stimulated with TLR ligands but not treated with cytochalasin D. Neither cytochalasin D nor LY294002 pretreatments had an effect on the level of *Il-6* transcript levels (Fig 2A), whereas both treatments significantly reduced levels of *Ifnβ* in response to purified curli-DNA complex. These data suggest that endocytosis of curli is required to produce a type I IFN response (Fig 2B). In support of this, in the presence of inhibitors of endocytosis, we observed decreases in *Ifnβ* transcript levels in IMMs stimulated with TLR9 ligands CpG or STM genomic DNA (Fig 2B).

We next investigated the uptake of curli by macrophages by measuring the relative fluorescent units (RFU) in wild-type and TLR2^-/-^ macrophages stimulated with 10 μg/mL of curli-DNA complex labeled with the amyloid specific dye Congo red [45]. After 1 hour of incubation with labeled curli, cells were washed with PBS to remove any extracellular Congo red-labeled curli-DNA complex and then lysed. Wild-type macrophages showed high levels of fluorescence, indicating an efficient phagocytosis; in contrast, in the absence of TLR2, we observed significantly lower levels of fluorescence. When the internalization of TLR2 was blocked using Cytochalasin D, RFU was decreased to background levels in wild-type macrophages suggesting that the uptake of curli is receptor-mediated and mainly dependent on TLR2 (Fig 2C). No signal due to labeled curli was detected in TLR2^-/-^ macrophages. We did not see an increase in the RFU in non-lysed cells (S3 Fig). The experiment was repeated using recombinant CsgA. Congo red-labeled His-CsgA was efficiently internalized into wild-type macrophages but not into TLR2^-/-^ macrophages (S3 Fig). To ensure that Hoescht 33258 specifically bound to the DNA portion of the curli-DNA complexes, we stained His-CsgA and His-CsgA fibrillized in the presence of 10 ng/ml CpG with 1 μg/ml of Hoescht 33258. We observed binding of Hoescht 33258 to the His-CsgA/DNA but not to the His-CsgA samples (S3 Fig).

To confirm the presence of curli within the endosomes containing TLR9, we treated macrophages expressing TLR9-GFP with Congo red-labeled curli-DNA complex and with LysoTracker blue, a dye that accumulates in endosomes. After 10 minutes of stimulation, the co-localization of TLR9 and curli-DNA complex within the endosome was evident by confocal microscopy with sequential scanning (Fig 2D).

### Curli complexes with DNA activate TLR9

The data above shows that activation of TLR9 and induction of type I IFNs by the curli-DNA complex requires activation of TLR2. Interestingly, curli-DNA complexes triggered production of higher levels of *Ifnβ* transcript than did CpG oligonucleotides or STM genomic DNA (Fig 2B). Antimicrobial peptides such as LL37 and defensins can amplify type I IFN production by binding DNA and forming complexes that potently activate TLR9 [46]. Recent work has shown that a key determinant for maximal TLR9-dependent interferon secretion is the inter-DNA spacing within the protein-DNA complexes. Inter-DNA spacings that match the steric size of TLR9 receptors (3.0–4.0 nm) allow for multivalent presentation of DNA ligands to TLR9, driving receptor recruitment, binding amplification, and immune activation [46]. We therefore measured the inter-DNA spacing within curli-DNA complexes.

**Fig 3:**
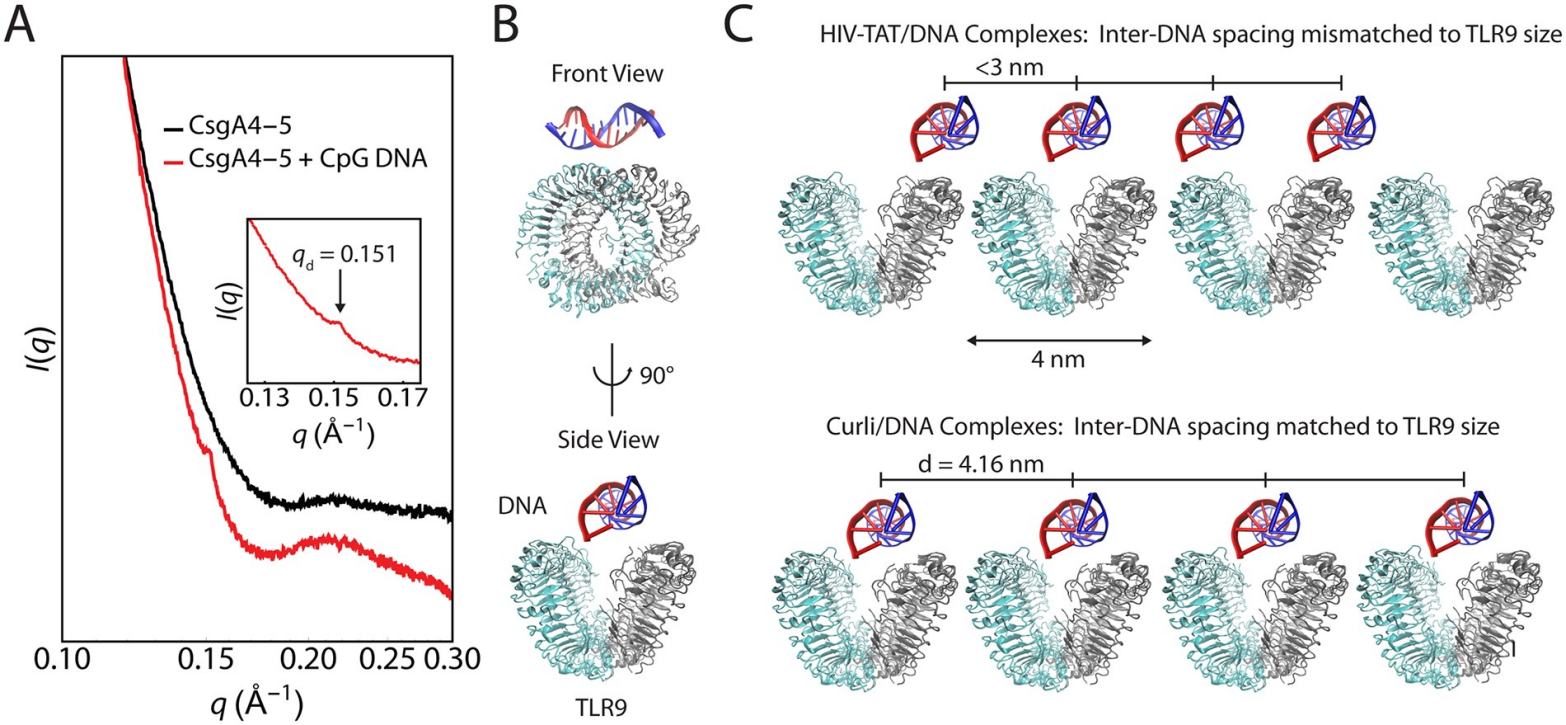
The structure of CsgA_R4-5_-DNA complexes explains their ability to induce type I interferon responses via TLR9. **A**. SAXS diffraction profiles for CsgA_R4-5_ amyloid fibrillized in the absence (black curve) and presence (red curve) of CpG dsDNA. No diffraction peaks indicating columnar structure are observed for the CsgA_R4-5_ fibrils in absence of DNA, but fibrillization of CsgA_R4-5_ in the presence of DNA results in a sharp diffraction peak at *q*d = 0.151 Å^-1^ (inset). This indicates that CsgA_R4-5_ organizes the CpG DNA into an ordered columnar lattice with inter-DNA spacing *d* = 2π/*q*_d_ = 4.16 nm. **B**. Schematic depicting CpG DNA binding to TLR9 from two orthogonal views. The anionic DNA binds electrostatically to the cationic paddles of TLR9. **C**. Closest approach of adjacent TLR9 receptors on the endosomal membrane does not allow size-matching and multivalent binding to HIV-TAT/DNA complexes (top). This data was adopted from Schmidt et al [46]. In contrast, the inter-DNA spacing for CsgA_R4-5_-DNA complexes (bottom) allows for amplification of binding from multivalent and electrostatic effects.

To determine the structure of curli-DNA complexes, we used a synthetic peptide corresponding to the fourth and fifth repeats of CsgA (CsgA_R4-5_) [18, 23]. The peptide was fibrillized in the presence or absence of CpG DNA. We measured the inter-DNA spacing within CsgA_R4-5_-DNA complexes using synchrotron small-angle X-ray scattering (SAXS). In the scattering profiles, we observed a sharp diffraction peak at 0.151 Å^-1^ for the CsgA_R4-5_-DNA complex but no significant scattering for the CsgA_R4-5_ alone (Fig 3A). We found that CsgA_R4-5_ organizes CpG DNA into an ordered columnar array that interacts with TLR9 at an inter-DNA spacing of 4.16 nm (Fig 3B). Although this value of inter-DNA spacing is slightly larger than the optimal inter-DNA spacings for interaction with TLR9 of 3.0–4.0 nm, it results in potent amplification of interferon production. Although the CsgA_R4-5_-DNA diffraction features were weak, the SAXS data shows clear diffraction features that are isotropic, indicating the lack of any preferred orientation in the sample. Furthermore, significance testing via statistical bootstrapping employing the maximal information coefficient and distance correlation analysis yielded P-values between P = 0.006 and P = 0.0157, indicating the existence of a strong and significant nonlinear relationship between TLR9 activation and inter-DNA spacing within DNA complexes. Thus we concluded that by organizing DNA into a columnar lattice with an inter-DNA spacing compatible with the steric size of TLR9, curli can maximize TLR9 binding to DNA, leading to the amplified type I IFN response observed (Fig 3C, bottom). Other peptides, such as HIV-TAT, are capable of endosomal access but do not efficiently activate TLR9 [46] because they organize DNA into lattices with inter-DNA spacings significantly smaller than 3 nm, which does not allow effective contact with the cationic domains of TLR9 (Fig 3C, top). Finally, our data suggests that CsgA_R4-5_ amyloid fibrillized in the absence of DNA does not form the ordered columnar lattices that are observed in the CsgA_R4-5_-DNA complex (Fig 3A). This emphasizes that fibrillization of CsgA_R4-5_ in the presence of DNA is required for its immunogenic activity.

### Deficiency in TLR2, TLR9, or both leads to attenuated production of autoantibodies and type I interferon response in vivo

Curli-expressing bacteria or injection of curli-DNA complexes into mice leads to the generation of anti-dsDNA antibodies [23]. As we determined that TLR2 and TLR9 are necessary for the response to curli-DNA complexes *in vitro*, we sought to investigate the role of these innate immune receptors in the generation of autoantibodies *in vivo*. To generate mice deficient in both TLR2 and TLR9, TLR2^-/-^ mice (B6.129-TLR2^tm1kir^/J) were mated with TLR9 mutant mice (C57BL/6J-Tlr9^M7Btlr^/Mmjax generated by Bruce Beutler) in house. We will refer to these as TLR2/TLR9-deficient. We injected wild-type (C57BL/6) mice, TLR2^-/-^ mice, TLR9 mutant mice, and TLR2/TLR9-deficient mice twice a week with 50 μg of purified curli-DNA complexes from *S*. Typhimurium *msbB* over a 6-week period. Serum samples were collected weekly and the concentration of anti-dsDNA autoantibodies were determined by ELISA. Serum from both untreated C57BL/6 mouse and PBS injected mouse were used as negative controls.

**Fig 4:**
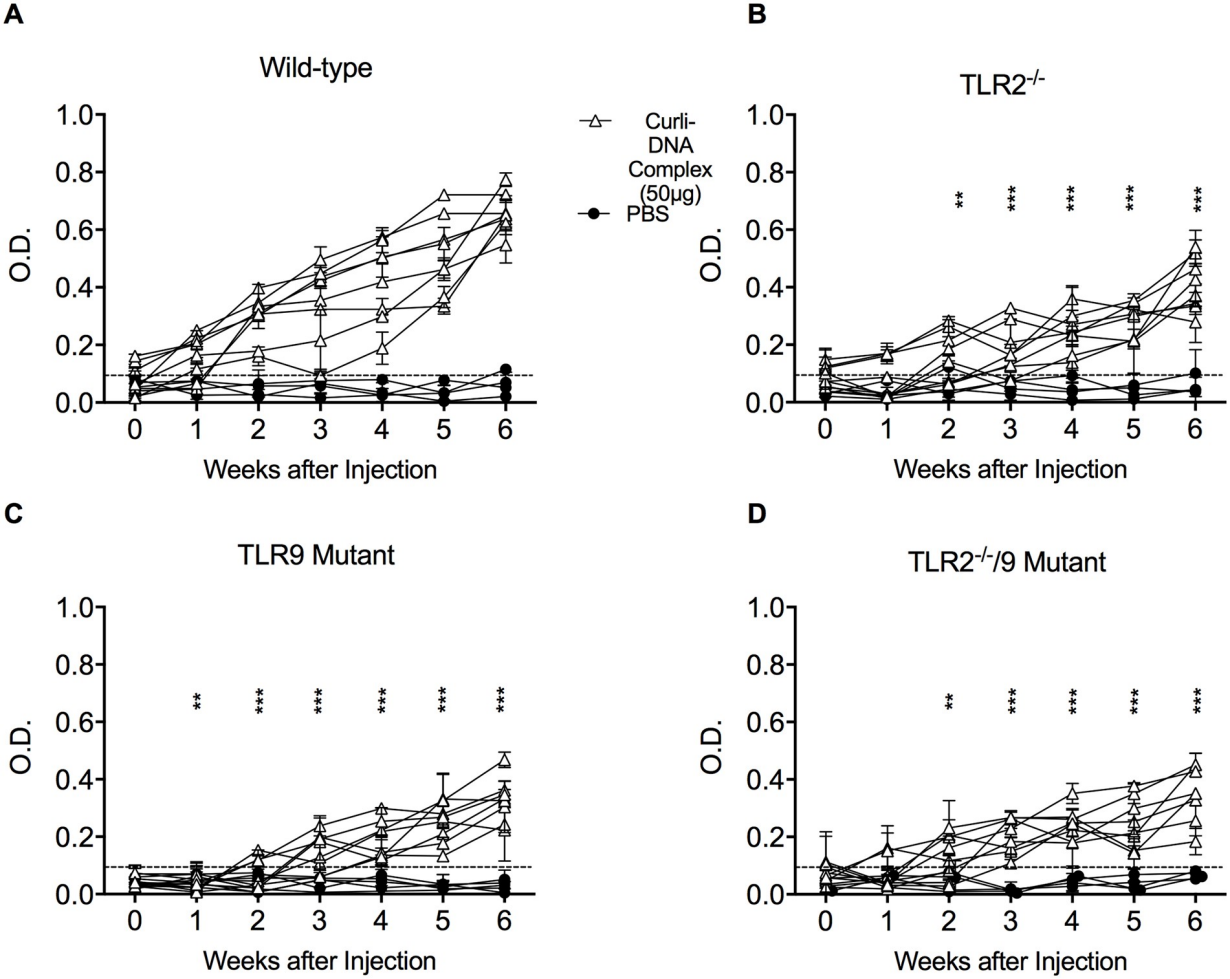
Curli-DNA complex leads to a delayed autoantibody response in TLR deficient mice. **A**) wild-type, (**B**) TLR2^-/-^ mice, (**C**) TLR9 mutant or (**D**) TLR2/TLR9-deficient mice were intraperitoneally injected with sterile PBS (circles and gray lines) or 50 μg of curli fibers isolated from *S*. Typhimurium IR715 *msbB* dissolved in sterile PBS (open triangles and black lines) twice weekly for 6 weeks. Each week serum was collected, and anti-dsDNA autoantibody quantified by ELISA. Serum collected from a naïve wild-type mouse was used as a negative control (dotted line). Mean and SE were calculated by averaging results from three independent experiments. Significance was calculated using Two-way Anova analysis followed by Tukey post hoc test. *p < 0.05, **p < 0.01, ***p < 0.001.

Wild-type mice injected with curli-DNA complexes began to develop anti-dsDNA autoantibodies after the first week, and the levels of autoantibodies increased over the 6-week period (Fig 4A). Wild-type mice injected with PBS did not develop autoantibodies. TLR2^-/-^, TLR9 mutant, and TLR2-TLR9-deficient mice injected with curli-DNA complexes had very low levels of autoantibodies during the first 3 weeks of the study (Fig 4B–4D). TLR2^-/-^ and TLR9 mutant mice began to develop autoantibodies after 4 weeks of curli-DNA injections. After 6 weeks of injections anti-dsDNA autoantibody levels were modest, and they remained significantly lower than the levels observed in wild-type mice (Fig 4C and 4D). Finally, the TLR2/TLR9-deficient mice developed a very low autoantibody response during the all 6 weeks of observation (Fig 4D). These clearly indicate a role for TLR2 and TLR9 in the development of autoantibodies against ds-DNA stimulated by curli-DNA complexes.

**Fig 5:**
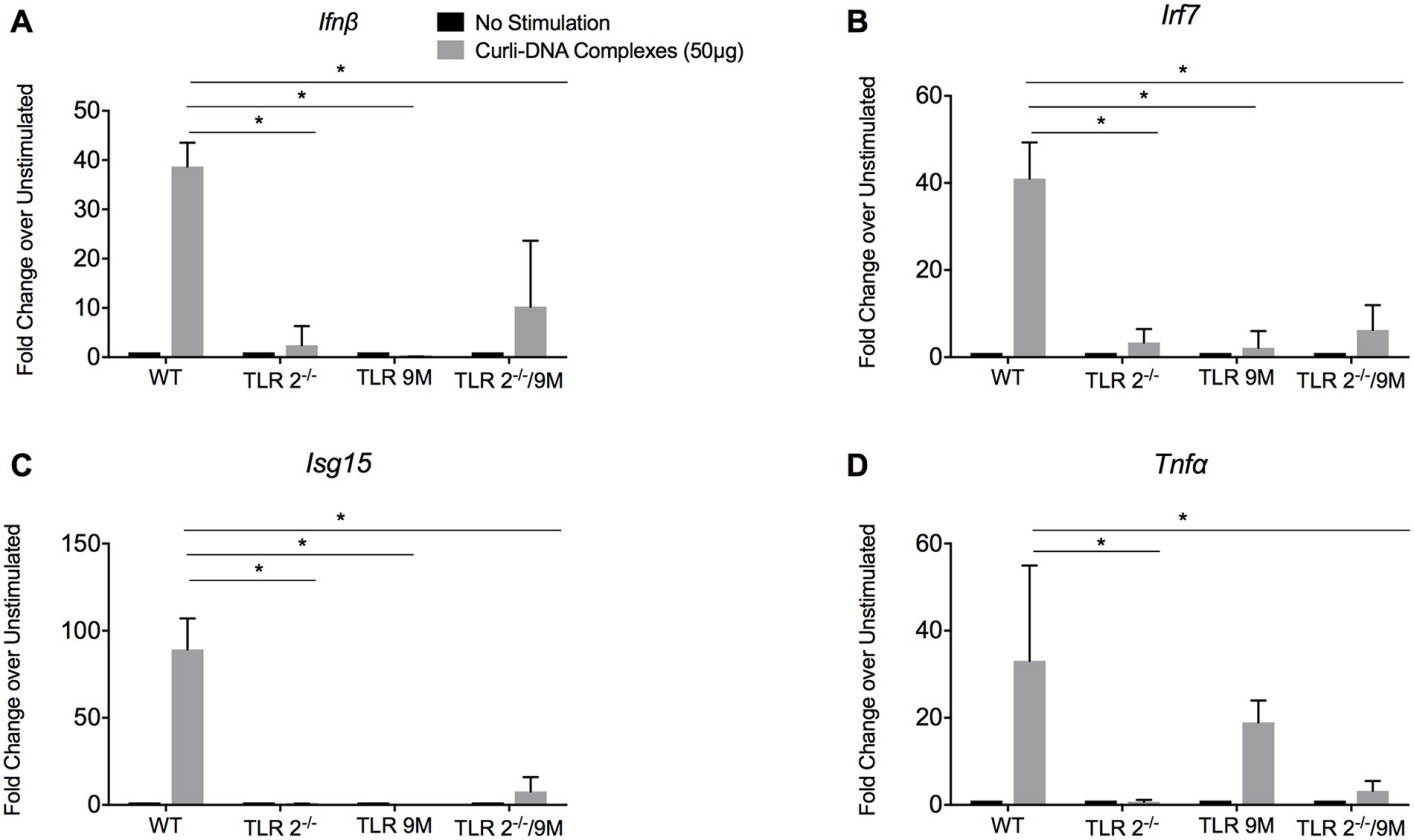
TLR9 activation by curli-DNA complex leads to a type I interferon response *in vivo*. Wild-type, TLR2^-/-^ mice, TLR9 mutant or TLR2/TLR9-deficient mice were intraperitoneally injected with sterile PBS or 50 μg of purified curli-DNA dissolved in sterile PBS. Mice were euthanized 4 hours after injection, and peritoneal lavage fluid was collected. (**A**) *Ifnβ*, (**B**) *Irf7*, (**C**) *Isg15*, and (**D**) *Tnfα* mRNA expression were quantified by qPCR. Mean and SE were calculated by averaging results from three independent experiments. Significance was calculated using students t-test. *p < 0.05, **p < 0.01, ***p < 0.001.

Reports have suggested that type I IFNs contribute to the production of autoantibodies in lupus by stimulating autoreactive B cells and plasmablasts that secrete autoantibodies [47, 48]. To determine whether TLR2 and TLR9 lead to the production of type I IFN response *in vivo*, wild-type mice and mice deficient in the receptors were intraperitoneally injected with 50 μg of purified curli-DNA complexes. After 4 hours, we collected immune cells from the peritoneal cavity. Cells were treated immediately with TriReagent, and the transcript levels of type I IFN *Ifnβ*, ISGs *Irf7* and *Isg15*, and *Tnfα* were determined. We observed a strong up-regulation of *Ifnβ*, *Irf7*, and *Isg15* in the peritoneal cells from wild-type mice, whereas in the absence of TLRs 2 and 9, we observed a significant decrease in the expression of these genes, suggesting that TLR2 and TLR9 are required for the *in vivo* IFN response (Fig 5A–5C). The level of *Tnfα*, a proinflammatory cytokine that is not a type I IFN responsive gene [38] was decreased relative to levels in wild-type mice only in TLR2^-/-^ and TLR2/TLR9-deficient mice (Fig 5D). No differences in the expression of *Tnfα* were detected in TLR9-deficient mice in comparison to wild-type mice (Fig 5D). These data are consistent with our *in vitro* findings and suggest that TLR2 is a primary PRR for curli-DNA and that this interaction induces NF-kB-dependent responses. Our data also show that both TLR2 and TLR9 are required for the stimulation of the type I IFN response *in vivo* induced by curli-DNA complexes.

### Cellulose does not contribute to the autoimmune response generated against curli-DNA complexes

**Fig 6:**
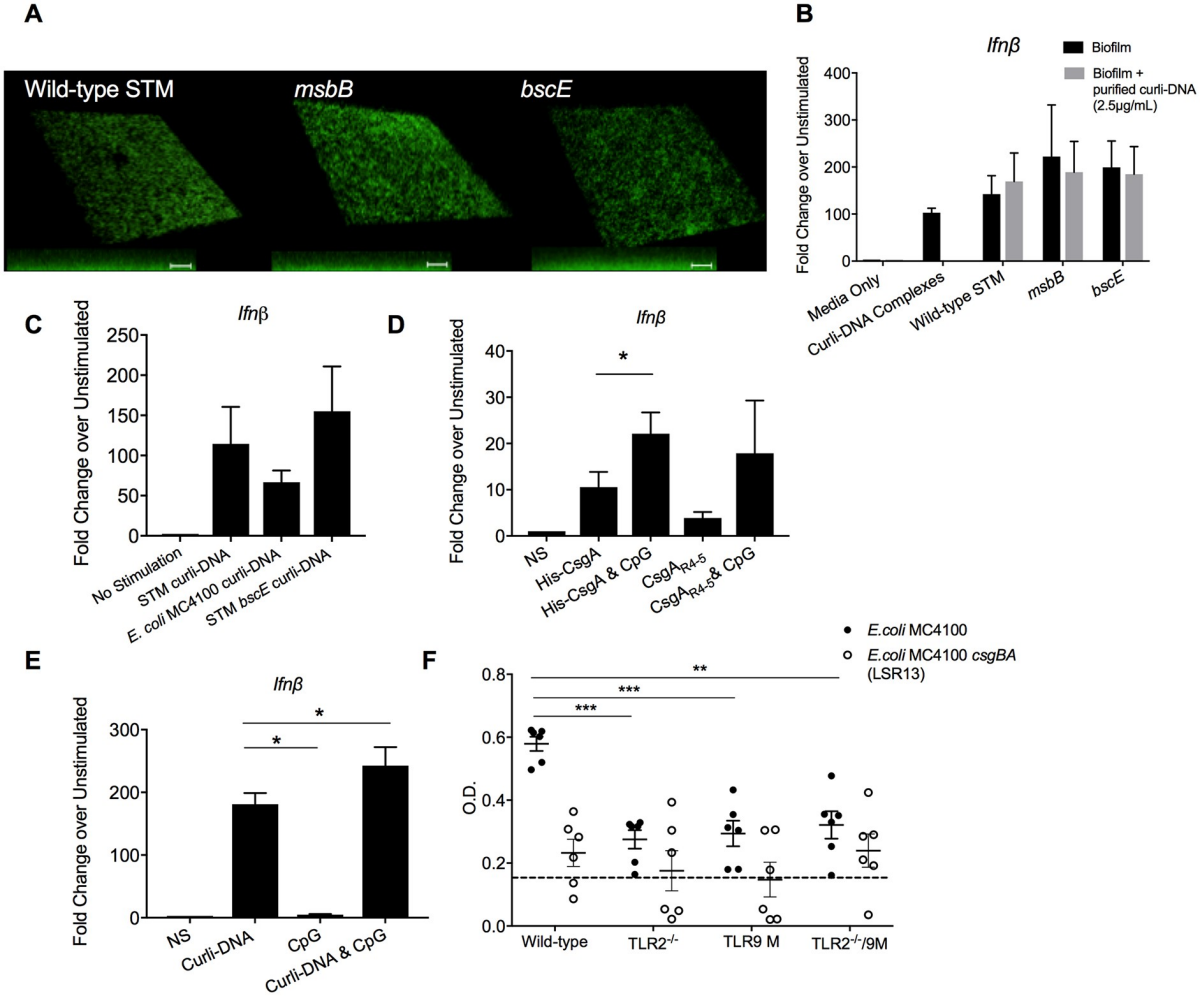
Curli and DNA contribute to the IFN response, but cellulose does not. **A**. Biofilms of *S*. Typhimurium wild-type IR715, *msbB* (LPS mutant), and *bscE* (cellulose mutant) were grown on glass coverslips in 24-well tissue culture dishes for 72 hours at 28°C. Biofilms were stained with 3 μg/ml Syto9 and visualized by microscopy (63x). 3D reconstructions were made using ImageJ software. **B**. Wild-type BMDCs were layered on top of established biofilms of wild-type *S*. Typhimurium, *msbB* and *bscE*. After 3 hours, *Ifnβ* was quantified in BMDCs by qPCR. As indicated, 2.5 μg/ml of curli-DNA complexes were layered on top of the biofilms prior to adding BMDCs. **C**. Wild-type IMMs (1x10^5^ cells per well) were stimulated with 2.5 μg/mL of curli-DNA complexes purified from *S*. Typhimurium, *E*. *coli* MC4100, or *S*. Typhimurium *bsc* for 3 hours, and *Ifnβ* was quantified by qPCR. **D**. Wild-type IMMs (1x10^5^ cells per well) were stimulated with 2.5 μg/ml of His-CsgA, His-CsgA polymerized in the presence of 10 ng/ml CpG DNA, synthetic peptide CsgA_R4-5_, or CsgA_R4-5_ polymerized in the presence of 10 ng/ml CpG DNA. After 3 hours, *Ifnβ* was quantified by qPCR. **E**. Wild-type IMMs (1x10^5^ cells per well) were stimulated with 2.5 μg/mL curli-DNA complexes, 10 ng/mL CpG DNA, or 2.5 μg/ml curli-DNA complexes and 10 ng/ml CpG. After 3 hours, *Ifnβ* was quantified by qPCR. Error bars indicate means ± S.E.M. from three independent experiments, * p <0.05 by students t-test. **F**. Wild-type, TLR2^-/-^, TLR9 mutant, and TLR2/TLR9-deficient mice were intraperitoneal injected with 1x10^5^ curli-expressing *E*. *coli* MC4100 (closed circles) or *E*. *coli* MC4100 *csgBA* (LSR13) (open circles) once weekly for 6 weeks. Serum collected from a naïve wild-type mouse was used as a negative control (dotted line). dsDNA autoantibodies were quantified by ELISA after 6 weeks of injections. Mean and SE were calculated by averaging results from two independent experiments. Significance was calculated using Two-way Anova analysis followed by Tukey post hoc test. *p < 0.05, **p < 0.01, ***p < 0.001.

Cellulose is another major component of the enteric biofilm extracellular matrix [49, 50]. Interactions between curli and cellulose leads to a honey-comb like structure in the matrix; however, cellulose and curli exhibit differential distribution and specific spatial arrangements in *E*. *coli* biofilms [51]. To ensure that the observed type I IFN and autoantibody response was due to curli-DNA complexes and not to cellulose, we grew biofilms of wild-type *S*. Typhimurium, the *msbB* mutant (which expresses a modified LPS that does not interact with TLR4), and a *bscE* mutant that does not express cellulose. At the 48 hour time point, all bacterial strains produced a similar biofilm mass (Fig 6A). Bone-marrow dendritic cells (BMDCs) were layered on top of established biofilms for 3 hours. BMDCs were recovered, and *Ifnβ* mRNA was quantified. There was no significant difference in levels of *Ifnβ* between the BMDCs exposed to different biofilm strains. *Ifnβ* transcript levels of BMDCs were also similar when biofilms were exogenously supplied with purified curli-DNA complex (Fig 6B). Bacteria that produce cellulose can be visualized by adding calcofluor-white to media. Whereas colonies of wild-type *S*. Typhimurium bind calcofluor-white, the *bcsE* mutant did not (S4 Fig). We also chose *E*. *coli* strain MC4100 that does not produce cellulose [33] (S4 Fig). When wild-type IMMs were stimulated with curli-DNA complexes purified from wild-type *S*. Typhimurium, *bcsE* mutant, or *E*. *coli* MC4100, there were no significant difference in the expression of *Ifnβ* (Fig 6C) or *Irf7* (S4 Fig). These results show that cellulose does not contribute to the induction of type I IFNs by curli-DNA complex.

Presently, it is technically impossible to purify the curli fibers without DNA or to remove DNA from curli fibers using current purification protocols. To interrogate the separate roles of curli and DNA, we used recombinant CsgA (His-CsgA) or synthetic peptide CsgA_R4-5_ polymerized in the presence or absence of CpG DNA. In wild-type IMMs treated with His-CsgA, His-CsgA/CpG, CsgA_R4-5_, or CsgA_R4-5_/CpG, we saw an increase in the expression of *Ifnβ* only when proteins were polymerized in the presence of CpG DNA (Fig 6D). When IMMs were treated with purified curli-DNA complex, exogenous addition of DNA also led to an increase in *Ifnβ* expression (Fig 6E).

To determine whether curli-DNA from cellulose-deficient bacteria can elicit anti-dsDNA autoantibody production *in vivo*, we injected wild-type, TLR2^-/-^, TLR9 mutant, and TLR2/TLR9-deficient mice with 10^5^ CFU of *E*. *coli* MC4100 or its isogenic curli-deficient *csgBA* mutant (LSR13) grown under curli-inducing conditions. Mice were injected with the same dose of bacteria every week for 6 weeks. We observed the development of anti-dsDNA autoantibodies in the wild-type mice injected with *E*. *coli* MC4100 but not with the *csgBA* mutant. TLR2^-/-^, TLR9 mutant, and TLR2/TLR9-deficient mice developed very low levels of anti-dsDNA antibodies (Fig 6E), resembling the response that we observed with the injection of purified curli-DNA complexes (Fig 4). Taken together, these data provide evidence that cellulose does not play a role in the stimulation of the type I IFN response and does not affect the autoantibody response to the curli-DNA complex. Moreover, these results confirm that curli-DNA complexes are the main auto-immunogens in bacterial infections by enteric bacteria.

## Discussion

The innate immune system is equipped with many PRRs that detect cognate conserved microbial signatures. In certain diseases, particular signatures dominate the clinical presentation. The prototypical example of such a dominant microbial structure is lipopolysaccharide (LPS), which governs the pathogenesis of Gram-negative septic shock. LPS is a particularly strong inducer of innate responses because it activates multiple PRRs, including complement, TLR4, and caspase-11 [52, 53].

Amyloids present in the bacterial biofilm matrix are another, but less extensively studied, microbial signature involved in the pathogenesis of bacterial infections. Like other bacterial PAMPs such as LPS and flagellin, amyloids are recognized by multiple innate immune receptors creating a unique immunological signature for biofilms. Our group and others have shown that amyloid fibers are recognized by TLR2/TLR1 heterocomplex as well as the NLRP3 inflammasome [16, 18, 22, 54–56]. Intriguingly, eukaryotic amyloids form complexes with DNA [57]. Bacterial amyloids curli and phenol-soluble modulins also bind to DNA [23, 58]. Here, we show that curli-DNA complexes create a type I IFN signature through the activation of TLR2 and TLR9. Cellulose was previously reported to reduce the production of IL-8, a proinflammatory cytokine, in response to uropathogenic *E*. *coli in vitro* [59]. However, cellulose does not contribute to the induction of type I IFNs or the anti-dsDNA autoantibodies by curli-DNA complexes (Fig 6). This suggests that more variable polysaccharide structures may not serve the same type of signaling functions as the possibly more conserved curli-DNA complexes. Activation of TLR9 by the DNA bound to curli increases the number of contributors that participate in the immune recognition of enteric biofilms. The presence of curli like amyloids and extracellular DNA in biofilms from multiple species also suggests that curli-DNA complexes may serve as a common immunological signature for biofilms.

Recently, our group uncovered a link between bacterial infections and the autoimmune disease systemic lupus erythematosus [23]. Patients with underlying autoimmune diseases or disorders suffer from frequent bacterial infections due to immunosuppressive therapies. Interestingly, SLE patients are particularly susceptible to enteric bacterial infections [60, 61]. We demonstrated that curli-DNA complexes found in enteric bacterial biofilms or infection with curli-expressing bacteria accelerate the progression of SLE via the generation of antibodies to dsDNA and a type I IFN response in murine models [23].

Type I IFNs are known to be associated with viral infections. Type I IFNs were initially discovered in 1950 for their ability to inhibit influenza infection in cell culture [62]. Upon infection with viruses, including Epstein-Barr, HIV, and norovirus, type I IFNs act to directly inhibit the spread of the virus [63–65]. Recent work showed that type I IFNs are also generated in response to bacterial infections, although the mechanisms by which the response influences infection remains unclear [66–68]. In this study, we elucidated the novel mechanism by which curli-DNA complexes trigger autoimmune responses via engagement of two important innate immune receptors, TLR2 and TLR9 (Figs 1–5). Only few reports have studied the role of TLR2 in lupus pathogenesis, focusing mostly on the damaging effects of lipoproteins in the kidney [69, 70]. Intriguingly, TLR2 drives the internalization of the curli-DNA complex into endosomes by binding to the structural motif of curli (Fig 2). The activation of TLR9 by DNA was previously shown to be a critical step in SLE pathogenesis [31], even though DNA by itself is a poor stimulator of the immune system [71, 72]. Human peripheral blood mononuclear cells, B cells, and pDCs can be activated by SLE sera or immune complexed self-DNA in part through TLR9 [73, 74]. Lupus-prone mice deficient in TLR9 do not develop anti-dsDNA autoantibodies, although other disease markers are increased [75, 76]. Here, we show that when DNA is complexed with the bacterial amyloid curli, it becomes a potent immunogen. Curli not only acts as a carrier to bring in the DNA into the cell and into endosomes but also organizes DNA at a spacing that potently activates TLR9 (Fig 3). Thus, we conclude that TLR2-dependent internalization of curli is an important first step for inducing high levels of subsequent aberrant immune activation via TLR9. Mice deficient in TLR2 and TLR9 mice had much lower levels of autoantibody production in response to curli-DNA than did wild-type mice (Fig 4). Overall, these results suggest that TLR2 and TLR9 are a new couple of pathogenic receptors involved in lupus progression that can be tested as therapeutic targets. We have previously reported that curli-DNA complexes stimulate the inflammasome [22]. The role of NLRP3 inflammasome activation in the autoimmune response is yet to be elucidated. It is possible that NLRP3-driven cell death also contributes to inflammation and generation of autoantigens, and may be responsible for the low levels of autoantibodies detected in the TLR2 and TLR9 mutant strains of mice.

Although many proteins can complex with DNA, not all these interactions result in structures that lead to receptor engagement. For instance, HIV-TAT peptide and human β-defensin-3 (HBD3) are both polycationic peptides that can penetrate cells [77] and gain access to endosomes where TLR9 resides. Incubation of human plasmacytoid DCs with TAT-DNA complexes does not produce significant levels of IFN-α, whereas HBD3-DNA complexes induce strong IFN-α production [77]. This suggests that endosomal access alone is not sufficient to trigger high levels of TLR9 activation. Our structural analysis suggests that the curli fibers organize DNA into an ordered columnar lattice with an inter-DNA spacing that promotes multivalent TLR9 binding and activation (Fig 3). Antimicrobial peptide-DNA complexes engage the TLR9 leading to high levels of type I IFN production [77]. However, curli-DNA complexes are expected to be qualitatively different from complexes formed between cationic antimicrobial peptides and anionic DNA. In contrast, curli and DNA are both strongly anionic, so the formation of complexes appears counterintuitive. Extensive recent work in polyelectrolyte physics, however, has shown that attractions between like-charged polymers (such as curli and DNA) can occur in the presence of common divalent ions (ex: Ca2+ or Mg2+), due to counterion correlations [78] The formation of ordered structures between highly anionic biological polymers that result from these like-charge attractions has been observed in a broad range of homogeneous and heterogeneous polymeric systems, including actin-actin and actin-DNA complexes [78–81]. Overall, these observations using the partial synthetic peptide (CsgA_R4-5_) suggest that further detailed structural analysis of curli-DNA complexes are needed to reveal the optimal engagement of TLR9 and possibly TLR2.

Overall, these findings are important because biofilms, that contain amyloid and DNA, are associated with numerous infections including urinary tract infections, osteomyelitis, and wound infections, and an estimated 65% of nosocomial infections are a result of bacterial biofilms [11–13]. Our identification of the conserved bacterial factors, DNA and curli, that contribute to the type I IFN response and the development of autoantibodies provides insight not only to the pathogenesis of biofilm-associated disease but also shed light into the interplay between infections and complex human diseases like SLE.

## Materials and methods

### Bacterial strains and culture conditions

*Salmonella enterica* serovar Typhimurium strain IR715 is a fully virulent, nalidixic acid-resistant strain derived from the ATCC strain 14028 [82]. *S*. Typhimurium IR715 *csgBA* is a mutant strain derived from *S*. Typhimurium IR715 that contains an unmarked *csgBA* deletion [20]. *S*. Typhimurium IR715 *msbB* and *S*. Typhimurium IR715 *fliCfljB* mutants were previously described [83]. Bacteria growth media was supplemented with 50 μg/mL nalidixic acid and 100 μg/mL kanamycin when appropriate. MC4100 and the MC4100 isogenic *csgBA* mutant (LSR13) were kindly provided by Dr. Matthew Chapman (University of Michigan, Ann Arbor, MI). *bscE*, a cellulose mutant derived from the ATCC strain *Salmonella enterica* serovar Typhimurium 14028, was a gift of Dr. John Gunn (The Ohio State University, Columbus OH).

T-media was prepared by mixing 10 g tryptone, 15 g agar in 1 L of water. YESCA agar was prepared by mixing 1 g of yeast extract with 20 g agar in 1 L of water. After autoclaving, 10 g of sterile casamino acids were added [15].

### Macrophage culture

Macrophage cell lines derived from wild-type mice (NR-9456), TLR2^-/-^ mice (NR-9457), TLR9^-/-^ mice (NR-9569), and TLR2^-/-^/TLR9^-/-^ mice (NR-19976) were obtained from BEI Resources. IMMs were maintained in DMEM (Invitrogen) supplemented with 10% heat-inactivated fetal bovine serum (FBS, Life Technologies). TLR9-GFP-expressing wild-type IMMs were generously provided by Dr. Melanie Brinkmann (Helmholtz Centre for Infection Research, Braunschweig, Germany) [84] were grown in DMEM supplemented with 10% FBS, 2 mM glutamine, 50 μM beta-mercaptoethanol. All macrophages were grown in a humidified incubator at 37°C with 5% CO_2._

Bone marrow-derived macrophages were generated from 6- to 8-week-old female C57BL/6 wild-type, TLR2^-/-^ (B6.129-TLR2^tm1kir^/J), TLR9 mutant mice (C57BL/6J-Tlr9M7Btlr/Mmjax, obtained from Jackson Laboratories), and the cross between the TLR2^-/-^ and the TLR9 mutant mice (B6.129-TLR2^tm1kir^/J-C57BL/6J-Tlr9M7Btlr/Mmjax; referred to as TLR2/TLR9-deficient mice) generated in house with approval from Temple University Institutional Animal Care and Use Committee. Macrophages were differentiated as described previously [17]. Briefly, mice were sacrificed and the femurs were kept on ice-cold RPMI 1640 media until the bone marrow could be extracted. Femurs were cleaned of excess tissue and flushed with 10 mL RPMI 1640 using a 27½-gauge needle. A single-cell suspension was created by suctioning the bone marrow through an 18-gauge needle, and the suspension was centrifuged at 1000 rpm for 10 minutes. The pellet was resuspended in 10 mL of bone marrow macrophage media (RPMI 1640, 10% FBS, 30% L929 cell conditioned RPMI 1640 media, supplemented with antibiotic-antimycotic (GIBCO, 15240) and L-glutamine (GIBCO, 25030)). A 2-mL aliquot of the cell suspension was put into each of five petri dishes, each dish supplemented with 13 mL of bone marrow macrophage media. Cells were incubated for 3 days at 37°C in 5% CO_2_. After the three days, the media was aspirated and replaced with 10 mL of fresh bone marrow macrophage media. On day 7 of culture, the spent media was aspirated and cells were washed with 1x PBS. Cells were subjected to treatment with 0.05% Trypsin (GIBCO, 25200–056) and incubated at 37°C in 5% CO_2_ for 15 minutes. Trypsinized cells were collected in a single tube and then centrifuged for 10 minutes at 1000 rpm. For analysis of effects of biofilms, 1x10^6^ of the BMDCs were placed on top of attached biofilms for 4 hours. RNA from the cells was harvested to determine the transcript levels of *Ifnβ* by qPCR using SYBR Green (Life Technologies).

### Purification of curli-DNA

Curli-DNA complexes were prepared using the protocol previously described [85]. Briefly, overnight cultures were grown in LB with proper antibiotic selection with shaking (200 rpm) at 37°C. Overnight cultures were diluted 1:100 in YESCA broth with 4% DMSO to enhance curli formation [86], and grown in a water bath at 26°C for 72 hours with shaking (200 rpm). Bacterial pellets were collected and resuspended in 10 mM Tris-HCl, pH 8.0 and treated with 0.1 mg/mL RNase A (Sigma, R5502) from bovine pancreas, 0.1 mg/mL DNase I (Sigma, DN25), and 1 mM MgCl_2_ for 20 minutes at 37°C. The bacterial cells were broken by sonication (30% amplification for 30 seconds, repeated twice). Lysozyme (1 mg/mL, Sigma, L6876) was added, and the mixture was incubated at 37°C. After 40 minutes, 1% SDS was added, and samples were incubated for 20 minutes at 37°C with shaking (200 rpm). The fibers were pelleted by centrifugation (10000 rpm for 10 minutes at room temperature) and resuspended in 10 mL Tris-HCL, pH 8 and boiled for 10 minutes. A second digestion with RNase A, DNase I, and lysozyme, followed by boiling, was performed as described above. Next, the fibers were pelleted (10000 rpm for 10 minutes at room temperature). Samples were boiled in 2X SDS-PAGE buffer and run on a 12% running/3-5% stacking gel for 5 hours at 20 mA. The fibers that accumulated at the top of the gel were collected and washed three times with sterile water and then extracted by washing twice with 95% ethanol. The fibers were then sonicated at 30% amplitude for 30 seconds to disrupt any large aggregates. The concentration of the curli fibers was determined using BCA reagent according to the manufacturer’s protocol (Novagen, 71285–3). Unless otherwise noted, curli-DNA complex isolated form *S*. Typhimurium IR715 *msbB* mutant was used to stimulate immortalized macrophages and bone marrow-derived macrophages. Hoescht 33258 (ThermoFisher, H3569) was used at a concentration of 1 μg/ml and Thioflavin T (Sigma, T3516) was used a concentration of 10 μM to stain extracellular DNA and amyloids, respectively.

### Dendritic cell-biofilm interactions

Bone Marrow-Derived Dendritic Cells (BMDC) were generated as described previously [23] from 6 to 8 week old female C57BL/6 wildtype mice For analysis of effects of biofilms, 1x10^6^ of the BMDCs were placed on top of attached biofilms for 4 hours. RNA from the cells was harvested to determine the transcript levels of *Ifnβ* by qPCR using SYBR Green (Life Technologies, 4309155).

To generate the biofilms, overnight cultures of wild-type *S*. Typhimurium or *msbB* or *bscE* mutants were grown overnight at 37°C with shaking (200 rpm) with appropriate selection. Cultures were diluted 1:100 into LB No Salt, and biofilms were grown in 48-well tissue culture plates statically at 28°C for 72 hours on an incline to allow for attachment of pellicle biofilms to the bottom of the wells. In some experiments, 2.5 μg/ml of curli-DNA complexes were layered upon the biofilms, and the biofilms were spun at 5000 rpm for 10 minutes to ensure the curli-DNA complexes were layered onto the biofilm.

Biofilms were visualized by generating overnight cultures of wild002Dtype or mutant *S*. Typhimurium diluted 1:100 in LB No Salt. Cultures were grown at a 45° angle in 24-well dishes with sterilized circular cover slips (Fisher, 1254581) placed in the bottom of the wells (creating a removable surface to which the biofilms could attach) for 72 hours at 28°C. After 72 hours, the biofilms were washed three times with sterile PBS and then stained with 3 μg/mL Syto9 (Molecular Probes, S34854) for 15 minutes shielded from light, and again washed three times with sterile PBS. The biofilms were then inverted and adhered to a microscope slide. Z-stacks of the biofilms were taken using a Leica SP5 Microscope with a TCS confocal system at 63x magnification. Syto9 fluorescence was visualized at an excitation of 485 and emission of 498. 3D reconstructions were created using Fiji (Image J) Software.

### Stimulation and analysis of murine immortalized macrophages

Wild-type, TLR2^-/-^, TLR9^-/-^, and TLR2^-/-^/TLR9^-/-^ IMMs were seeded at 1x10^5^ per well in a 48-well polystyrene dish (Costar, 3524). Cells were stimulated with purified curli for 3 or 24 hours. The supernatant was collected and IL-10 and TNFα were quantified by ELISA according to manufacturer’s protocol (eBiosciences). To determine the effect of phagocytosis on type I IFN signaling, wild-type murine IMMs were seeded in a 48-well plate polystyrene dish (Costar, 3524) at 5x10^5^ cells per well. After 1 hour, cells were stimulated with 2.5 μM Cytochalasin D to block actin polymerization (Sigmal Aldrich, C2618) or 50 μM LY294002 (Cell Signaling) to inhibit PI3K. After 1 hour, cells were stimulated with 2.5 μg/ml purified curli for 3 hours.

To investigate the contribution of DNA in recognition of the curli-DNA complex, wild-type IMMs were stimulated with 2.5 μg/mL His-CsgA, 2.5 μg/mL His-CsgA polymerized with 10 ng/mL CpG DNA (Invivogen, ODN 1826), 2.5 μg/mL CsgA_R4-5_, 2.5 μg/mL CsgA_R4-5_ polymerized in the presence of 10 ng/mL CpG DNA (ODN 1826, Invivogen), 2.5 μg/mL curli-DNA complexes, 10 ng/mL CpG, and 2.5 μg/mL curli-DNA complexes. His-CsgA was reported previously [17] and purified using the BugBuster Ni-NTA His-Bind Purification Kit (EMD Millipore, 70751) according to manufacturer’s protocol.

To determine transcript levels of various proinflammatory and type I IFN regulated genes, RNA was extracted using TriReagent according to the manufacturer’s protocol (MRC, TR118). RNA was then treated with DNase according to the manufacturer’s protocol (Ambion, AM1906) and then reverse transcribed to cDNA using TaqMan Reverse Transcription kit according to manufacturer’s protocol (Invitrogen, N8080234). Transcript levels were determined by quantitative real time reverse transcription PCR (qPCR) using SYBR Green (Life Technologies, 4309155) using the ΔC_T_ approach. Primers used are listed in Table 1.

**Table 1:** Primers used for qPCR.

Gene Target	Direction	Sequence	Source
*Ifnβ*	Forward	5' CAG CTC CAA GAA AGG ACG AAC 3’	Harvard Primer Bank ID: 6754304a1
	Reverse	5' GGC AGT GTA ACT CTT CTG CAT 3’	Harvard Primer Bank ID: 6754304a1
*Irf7*	Forward	5' CAG CAG TCT CGG CTT CTG 3’	[87]
	Reverse	5' TGA CCC AGG TCC ATG AAG TG 3’	[87]
*Isg15*	Forward	5' GGT CTC CGT TAA CTC CAT 3’	Harvard Primer Bank ID: 7657240a1
	Reverse	5' TCC AAA GGG TAA CAC CGT CCT 3’	Harvard Primer Bank ID: 7657240a1
*Cxcl10*	Forward	5’ CCA AGT GCT GCC GTC ATT TTC 3’	Harvard Primer Bank ID: 10946576a1
	Reverse	5’ GGC TCG CAG GGA TGA TTT CAA 3’	Harvard Primer Bank ID: 10946576a1
*Tnfα*	Forward	5’ CCC TCA CAC TCA GAT CAT CTT CT 3’	[88]
	Reverse	5’ GCT ACG ACG TGG GCT ACA G 3’	[88]
*Il-6*	Forward	5’ GGTGCCCTGCCAGTATTCTC 3’	Harvard Primer Bank ID: 7110655a1
	Reverse	5’ GGCTCCCAACACAGGATGA 3’	Harvard Primer Bank ID: 7110655a1
*Gapdh*	Forward	5’ CCA GGA AAT CAG CTT CAC AAA CT 3’	[88]
	Reverse	5’ CCC ACT CCT CCA CCT TTG AC 3’	[88]

### Analysis of localization of TLR9 and curli-DNA complex within the macrophage endosome

To visualize TLR9 and curli, TLR9-GFP expressing macrophages were seeded at 1x10^6^ per well in a 48-well dish upon circular coverslips (Fisher, 1254581 coated with poly-L-lysine (Sigma, P8920). After attachment, macrophages were incubated with 75 nM LysoTracker Blue DND-22 (ThermoFisher, L7525) and 10 μg/mL Congo red-labeled curli-DNA complexes for 10 minutes at 37°C and 5% CO_2_. To label curli with Congo red, 1 mg/mL purified curli-DNA fibers were incubated with 50 μg/mL Congo red (Sigma, C6767) for 15 minutes protected from light. The curli fibers were pelleted by centrifugation at 12,000 rpm for 5 minutes. The pellet was washed with 500 μL sterile PBS five times, and then resuspended in 1 mL of sterile PBS. After 10 minutes of stimulation, cells were washed with sterile PBS three times and visualized using a Leica SP5 Microscope with a TCS confocal system using sequential scanning to prevent auto-fluorescence from multiple lasers. LysoTracker Blue DND-22 was visualized at an excitation wavelength of 373 nm and emission of 422 nm, and Congo red-labeled curli was visualized at an excitation wavelength of 514 nm and emission of 650–750 nm.

### Quantification of internalization of TLR2-bound curli-DNA complex

To quantify the internalization of TLR2-bound curli-DNA complex, wild-type and TLR2^-/-^ IMMs were seeded in a 48-well polystyrene plate (Costar, 3524) at 1x10^6^ cells per well. After 1 hour, cells were stimulated with 2.5 μM Cytochalasin D to block actin polymerization. Cells were stimulated with Congo red-labeled curli for 1 hour. Cells were washed three times with sterile PBS to remove any extracellular labeled curli-DNA complexes and then were lysed with sterile PBS supplemented with 1% Triton-X and transferred to a clear bottom, black, 96-well microplate for fluorescent readings. Fluorescence of Congo red-labeled curli was measured using Flex Station, Molecular Devices, at an excitation of 497 nm and emission at 614 nm.

### Small angle X-ray scattering

CsgA_R4-5_ was purchased from Biosynthesis Incorporated (Lot #T876-2) and dissolved in 500 μL hexafluoroisopropanol (HIFP) and concentrated by speedvac (Savant) for approximately 1 hour. The pellet was then resuspended in 500 μL DMSO. CsgA_R4-5_ monomers were then purified using a Hi-Trap Column according to manufacturer’s protocol (GE Healthcare, 17140701). CsgA_R4-5_ was fibrillized alone or in the presence of 10 ng/mL CpG DNA (Invivogen, ODN 1826). Fibrils with and without CpG DNA were pelleted in Eppendorf tubes with centrifugation at 10,000 rpm for 20 minutes. All but 50 μL of supernatant was discarded. The fibrils were resuspended in remaining supernatant and loaded and sealed in quartz capillaries (Mark-tubes, Hilgenberg, GmbH). The samples were stored at 4°C until measurement. SAXS experiments were performed at the Stanford Synchrotron Radiation Lightsource (SSRL, Beamline 4–2) using monochromatic X-rays with an energy of 9 keV. The scattered radiation was measured using a Rayonix MX-225-HE detector (pixel size 73.2 μm). 2D powder diffraction patterns were integrated using the Nika 1.74 [89] package for Igor Pro 6.37 and FIT2D [90]. SAXS data were analyzed by plotting integrated scattering intensity against the momentum transfer *q* using Mathematica. Peak positions were measured by fitting diffraction peaks to a Lorentzian. The inter-DNA spacing *d* was obtained from the first peak position *q*_*d*_ by the formula *a* = 2π/*q*_*d*_.

### Animal experiments

To determine the involvement of TLR2 and TLR9 in the recognition of curli-DNA complexes and generation of a type I IFN response, 6- to 8-week-old wild-type (C57BL/6), TLR2^-/-^ (B6.129-TLR2^tm1kir^/J), TLR9 mutant (C57BL/6J-Tlr9M7Btlr/Mmjax), and mice deficient in TLR2 and TLR9 (obtained by crossing B6.129-TLR2^tm1kir^/J and C57BL/6J-Tlr9M7Btlr/Mmjax) were intraperitoneally injected with 100 μL sterile PBS or 50 μg purified curli-DNA complexes in 100 μL sterile PBS using a 27-gauge needle. After 3 hours, lavage cells were collected by injecting 3 mL of pre-chilled PBS supplemented with 3% FBS into the peritoneal cavity and removing the cells using 25-gauge needle. RNA was collected from the extracted cells, and *Ifnβ*, *Irf7*, *and Isg15* were quantified using q-PCR as described above.

To determine the generation of autoantibodies upon administration of curli-DNA complexes, 6- to 8-week-old female wild-type (C57BL/6), TLR2^-/-^ (B6.129-TLR2^tm1kir^/J), TLR9 mutant (C57BL/6J-Tlr9M7Btlr/Mmjax), and mice deficient in TLR2 and TLR9 (B6.129-TLR2^tm1kir^/J-C57BL/6J-Tlr9M7Btlr/Mmjax) were intraperitoneally injected with 100 μl sterile PBS or 50 μg purified curli in 100 μL sterile PBS twice a week for 6 weeks. Each week, mice were tail bled to collect serum samples.

To investigate the role of cellulose in immune recognition of the curli-DNA complexes, overnight cultures of the cellulose lacking *E*. *coli* MC4100 and its isogenic *csgBA* mutant were grown on T-media for 28°C for 72 hours to induce biofilm formation. Bacterial cells (1x10^5^ cells per injection) or sterile PBS were intraperitoneally injected weekly for 6 weeks into 6- to 8-week-old female wild-type (C57BL/6), TLR2^-/-^ (B6.129-TLR2^tm1kir^/J), TLR9 mutant (C57BL/6J-Tlr9M7Btlr/Mmjax), and mice deficient in TLR2 and TLR9 (B6.129-TLR2^tm1kir^/J-C57BL/6J-Tlr9M7Btlr/Mmjax). Each week, mice were tail bled to collect serum samples.

### Anti-dsDNA autoantibody ELISA

This protocol was performed as previously described [23]. Briefly, a 96-well plate (Costar, 07-200-33) was coated with 0.01% poly-L-lysine (Sigma, P8920) in PBS for 1 hour at room temperature and washed three times with distilled water and dried. The plate was coated with 2.5 μg/mL calf thymus DNA (Invitrogen, 15633–019) in BBS (17.5 g NaCl, 2.5 g H_3_BO_3_, 38.1 g sodium borate in 1 L H_2_O) and stored overnight at 4°C overnight. The plate was washed three times with borate buffered saline (BBS) and blocked with BBT (BBS, 3% BSA, 1% Tween20) for 2 hours at room temperature. After washing five times with BBS, the plate was incubated with serial dilutions of control serum, naïve serum, or serum samples overnight at 4°C. After washing, the biotinylated goat anti-mouse IgG (Jackson ImmunoRes, 115-065-071) was added, and samples were incubated at room temperature for 2 hours with gentle rocking, and then incubated with avidin-alkaline phosphate conjugate (Sigma, A7294) at room temperature for 2 hours. Finally, the plate was washed five times with BBS and then incubated with 4-nitrophenyl phosphate disodium salt hexahydrate (Sigma-Aldrich, N2765) at a concentration of 1 mg/mL at room temperature protected from light. Optical densities were read using ELISA plate reader at 650 nm and 405 nm using a Molecular Devices Microplate Reader. Serum of a 6-to 8-week-old C57BL/6 mouse with no evidence of autoimmunity was used as a negative control for autoantibody production. As a positive control, a serum from an old B6.NZM Sle1/Sle2/Sle3 lupus-prone mice (Sle1,2,3 mice) previously tested for high levels of autoantibodies, was used as diluted 1:250 in BBST. All the samples shown in each figure were tested in the same ELISA assay, and the result are shown as raw Optical Density (O.D.).

### Statistical analyses

Data were analyzed using Prism software (GraphPad, San Diego). Two way Anova with post-hoc Tukey Multiple Comparison tests or two-tailed Student’s t test were used as appropriate. The *p* values <0.05 were considered significant. *p < 0.05, **p < 0.01, ***p < 0.001 were marked in the figures.

### Ethics statement

All animal experiments were performed in BSL2 facilities with protocols that are approved by AALAC-accredited Temple University Lewis Katz School of Medicine, Institutional Animal Care and Use Committee (IACUC# 4561) in accordance with guidelines set forth by the USDA and PHS Policy on Humane Care and Use of Laboratory Animals under the guidance of the Office of Laboratory Animal Welfare (OLAW). The institution has an Animal Welfare Assurance on file with the NIH Office for the Protection of Research Risks (OPRR), Number A3594-01.

## Supporting information

S1 Fig Purified curli-DNA complexes contain amyloids and DNA. **A**. Curli fibers purified from wild-type *S*. Typhimurium and *csgBA* and *msbB* mutants were stained with 1 μg/mL Hoescht 33258 (nucleic acid stain) and 10 μM Thioflavin T (amyloid stain). **B**. Concentration of DNA extracted from 1 mg purified curli-DNA fibers using a phenol-chloroform extraction procedure determined using a Nano-Drop (Thermofisher). **C**. Curl-DNA fibers purified from wild-type *S*. Typhimurium (top panel) and *msbB* mutant (bottom panel) using sequential differential centrifugation were stained with 1 μg/mL Hoescht 33258 (nucleic acid stain) and 10 μM Thioflavin T (amyloid stain). **D**. Immune responses of wild-type, TLR2^-/-^, TLR9^-/-^, and TLR2^-/-^/TLR9^-/-^ bone marrow-derived macrophages stimulated with 2.5 μg/mL of curli-DNA complexes (traditional purification method) or 2.5 μg/mL of curli-DNA complexes isolated by sequential differential centrifugation. Macrophages were stimulated for 3 hours, and the transcript levels of *Isg15* and *Ifnβ* were determined by qPCR. Bars represent means ± S.E.M. from at least two independent experiments, * p <0.05 as determined by Students t-test.
(TIFF)

S2 Fig Bone marrow-derived macrophages elicit a type I interferon response to curli-DNA complexes dependent on TLR9. **A**. Wild-type, TLR2^-/-^, TLR9^-/-^, and TLR2^-/-^/TLR9^-/-^ bone marrow derived macrophages (1x10^5^ cells) were stimulated with 2.5 μg/ml of *S*. Typhimurium IR715 *msbB* curli-DNA complexes for 3 hours, and *Ifnβ* was quantified by q-PCR. **B**. Levels of IL-6 at the 3-hour time point were also determined by ELISA. Bars represent means ± S.E.M. from at least three independent experiments, * p <0.05 as determined by Students t-test.
(TIFF)

S3 Fig Congo red-labeled curli binding to macrophages. **A**. Wild-type and TLR2^-/-^ macrophages (1x10^6^ cells per well) were stimulated for 1 hour with 10 μg/ml Congo red-labeled curli-DNA complexes. After 1 hour, cells were washed three times with sterile PBS and lysed with PBS supplemented with 1% triton-X or not lysed. Cells were transferred to black-walled optical 96-well plates, and RFU measured using Flex Station (Molecular Devices) at an excitation of 497 nm and an emission 614 nm. **B**. 1x10^6^ Wild-type TLR2^-/-^ macrophages (1x10^6^ cells pre well) were stimulated for 1 hour with 10 μg/ml Congo red-labeled His-CsgA. Cells were lysed with sterile PBS supplemented with 1% Triton-X and RFU was measured. **C**. His-CsgA and His-CsgA fibrillized in the presence of 10 ng/ml CpG was stained with 1 μg/ml Hoescht 33258, and fluorescence images were captured using an Olympus BX60 Fluorescent Microscope with Spot Insight2 camera. Bars represent means ± S.E.M. from at least three independent experiments, * p <0.05 as determined by Students t-test.
(TIFF)

S4 Fig Impact of cellulose on the type I IFN profile. **A**. Cellulose expression was visualized by spotting 5 μl of overnight culture of *S*. Typhimurium, *bscE mutant*, *E*. *coli* MC4100, or *E*. *coli* MC4100 *csgBA* (LSR13) on LB supplemented with calcofluor-white and grown at 28°C for 72 hours. Colonies were visualized using a transilluminator. **B**. *Ifnβ* was quantified after stimulation of 1x10^6^ wild-type macrophages with purified curli-DNA complexes purified from *S*. Typhimurium, *E*. *coli* MC4100, or *S*. Typhimurium *bscE* mutant for 3 hours. Bars represent means ± S.E.M. from at least three independent experiments, * p <0.05 as determined by Students t-test.
(TIFF)
