# Supplementary figures and images for: Bacterial amyloid curli acts as a carrier for DNA to elicit an autoimmune response via TLR2 and TLR9
Source: PLoS Pathog. 2017 Apr 14;13(4):e1006315. doi: 10.1371/journal.ppat.1006315 (PMC5406031; doi:10.1371/journal.ppat.1006315)

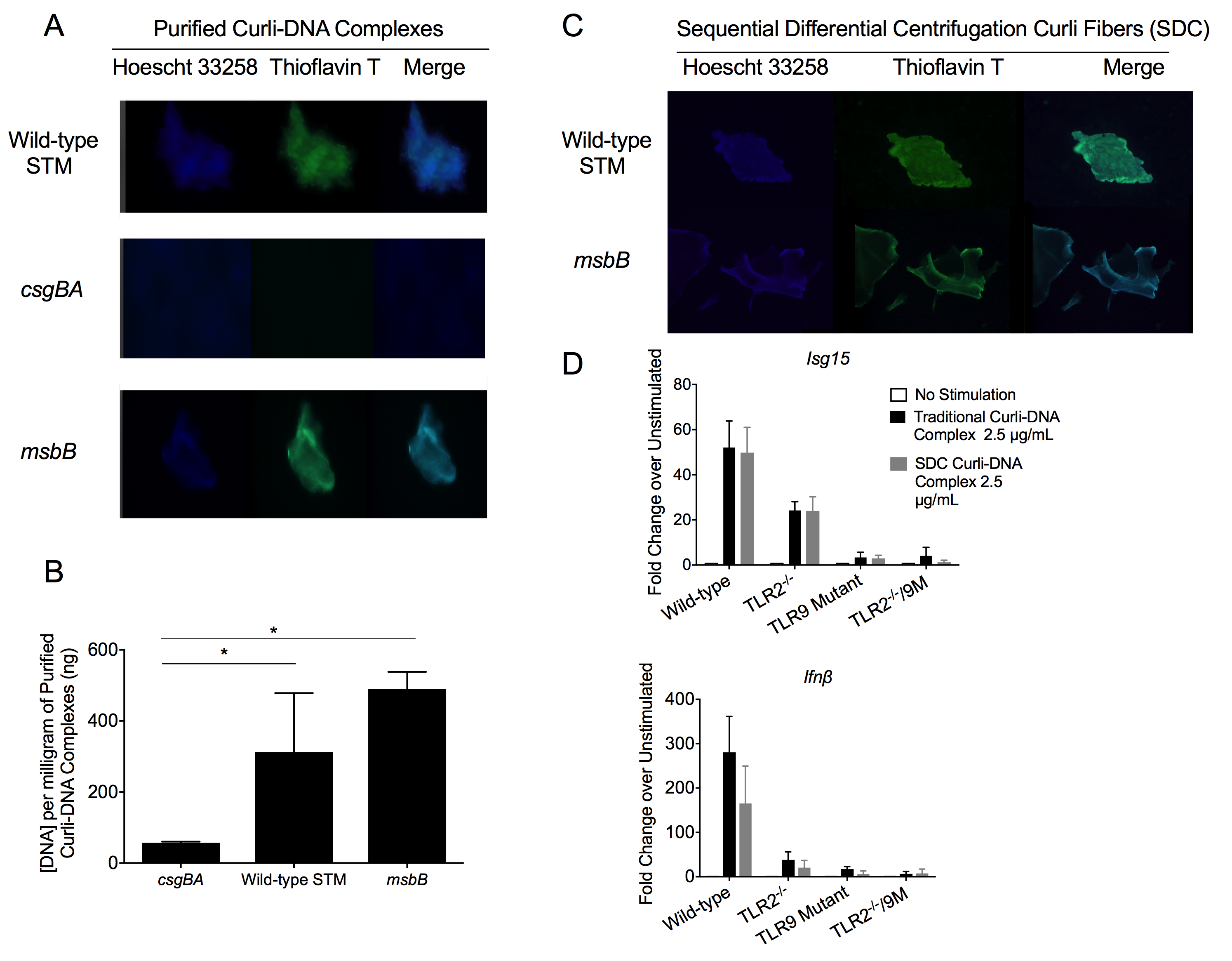

Supplement: S1 Fig — A. Curli fibers purified from wild-type S. Typhimurium and csgBA and msbB mutants were stained with 1 μg/mL Hoescht 33258 (nucleic acid stain) and 10 μM Thioflavin T (amyloid stain). B. Concentration of DNA extracted from 1 mg purified curli-DNA fibers using a phenol-chloroform extraction procedure determined using a Nano-Drop (Thermofisher). C. Curl-DNA fibers purified from wild-type S. Typhimurium (top panel) and msbB mutant (bottom panel) using sequential differential centrifugation were stained with 1 μg/mL Hoescht 33258 (nucleic acid stain) and 10 μM Thioflavin T (amyloid stain). D. Immune responses of wild-type, TLR2-/-, TLR9-/-, and TLR2-/-/TLR9-/- bone marrow-derived macrophages stimulated with 2.5 μg/mL of curli-DNA complexes (traditional purification method) or 2.5 μg/mL of curli-DNA complexes isolated by sequential differential centrifugation. Macrophages were stimulated for 3 hours, and the transcript levels of Isg15 and Ifnβ were determined by qPCR. Bars represent means ± S.E.M. from at least two independent experiments, * p <0.05 as determined by Students t-test. (TIFF) [file ppat.1006315.s001.tiff]

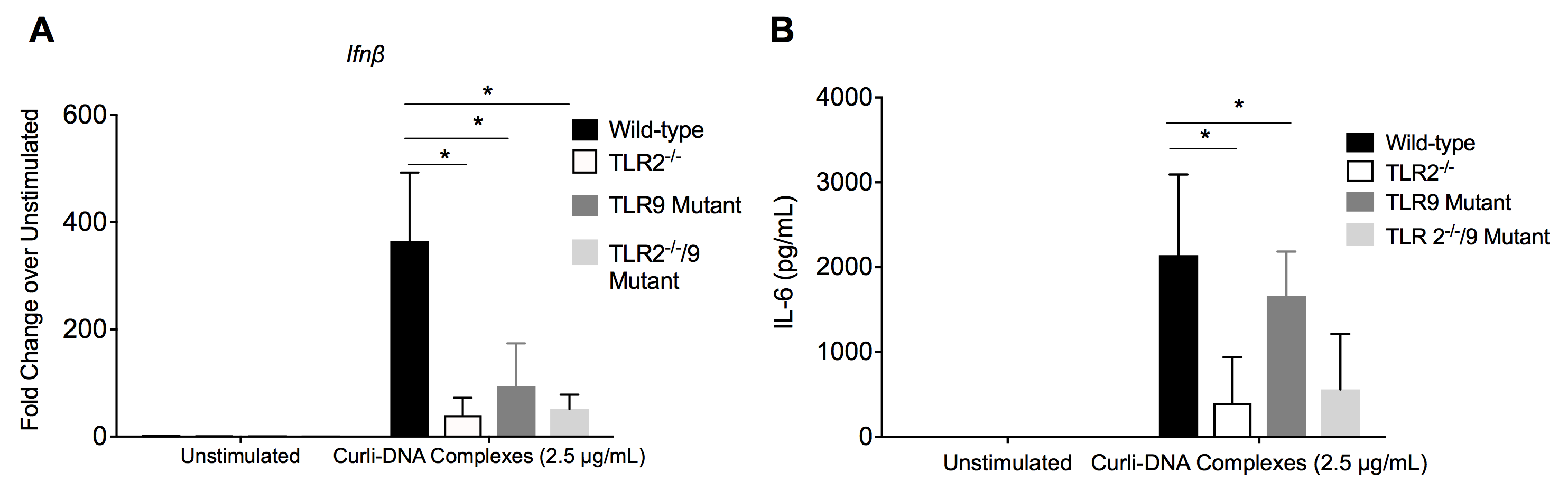

Supplement: S2 Fig — A. Wild-type, TLR2-/-, TLR9-/-, and TLR2-/-/TLR9-/- bone marrow derived macrophages (1x10^5^ cells) were stimulated with 2.5 μg/ml of S. Typhimurium IR715 msbB curli-DNA complexes for 3 hours, and Ifnβ was quantified by q-PCR. B. Levels of IL-6 at the 3-hour time point were also determined by ELISA. Bars represent means ± S.E.M. from at least three independent experiments, * p <0.05 as determined by Students t-test. (TIFF) [file ppat.1006315.s002.tiff]

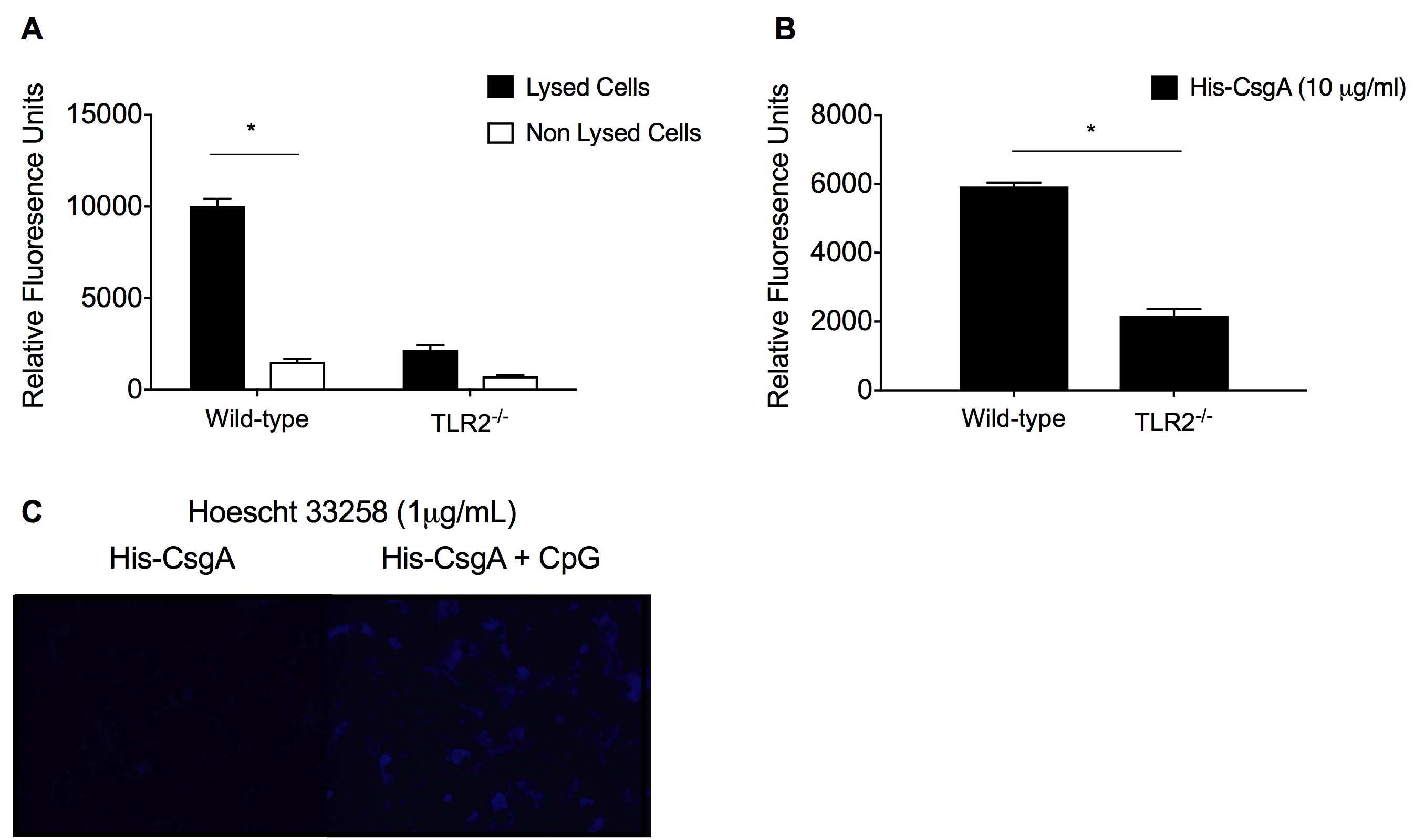

Supplement: S3 Fig — A. Wild-type and TLR2-/- macrophages (1x10^6^ cells per well) were stimulated for 1 hour with 10 μg/ml Congo red-labeled curli-DNA complexes. After 1 hour, cells were washed three times with sterile PBS and lysed with PBS supplemented with 1% triton-X or not lysed. Cells were transferred to black-walled optical 96-well plates, and RFU measured using Flex Station (Molecular Devices) at an excitation of 497 nm and an emission 614 nm. B. 1x10^6^ Wild-type TLR2-/- macrophages (1x10^6^ cells pre well) were stimulated for 1 hour with 10 μg/ml Congo red-labeled His-CsgA. Cells were lysed with sterile PBS supplemented with 1% Triton-X and RFU was measured. C. His-CsgA and His-CsgA fibrillized in the presence of 10 ng/ml CpG was stained with 1 μg/ml Hoescht 33258, and fluorescence images were captured using an Olympus BX60 Fluorescent Microscope with Spot Insight2 camera. Bars represent means ± S.E.M. from at least three independent experiments, * p <0.05 as determined by Students t-test. (TIFF) [file ppat.1006315.s003.tiff]

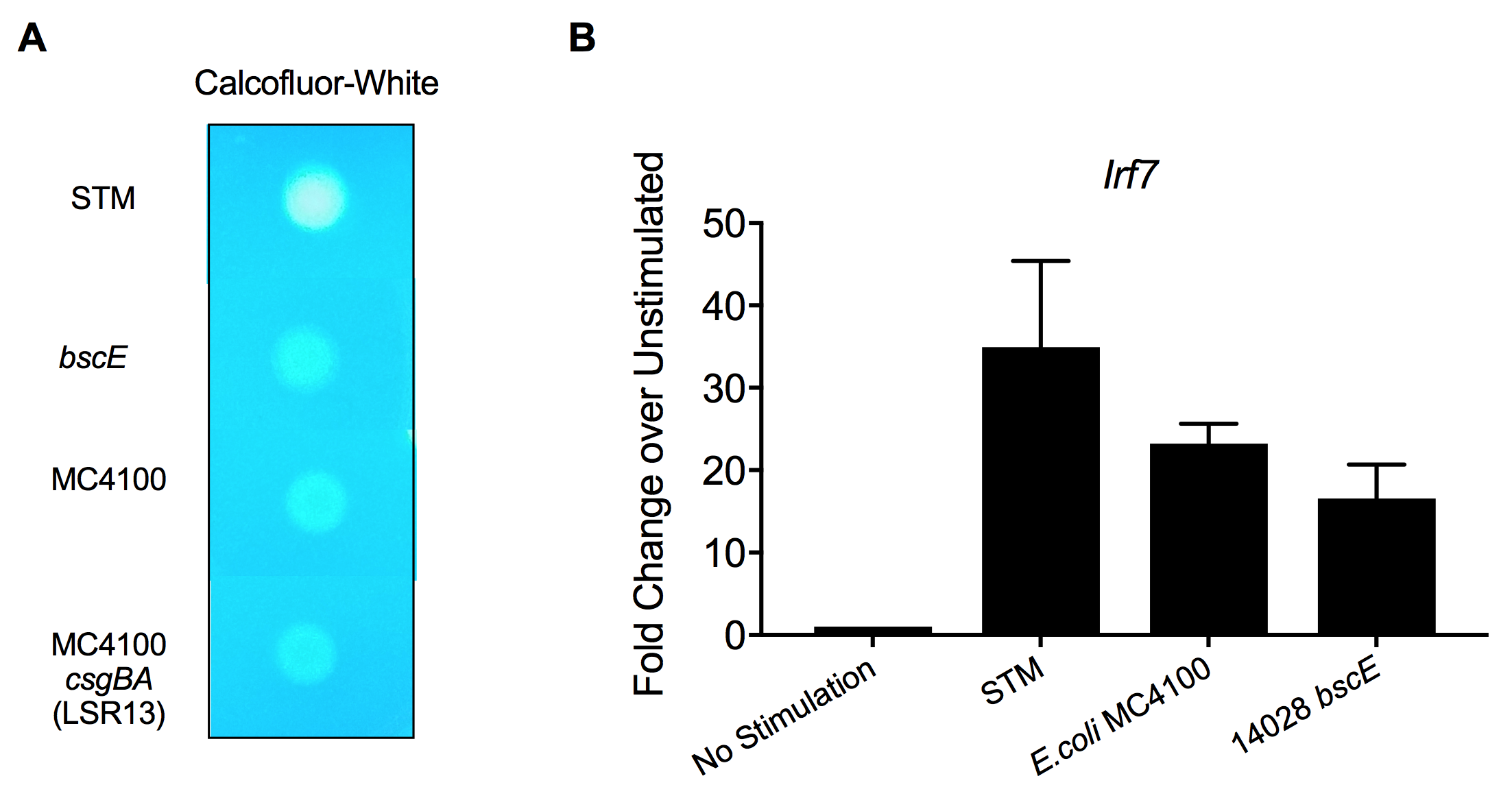

Supplement: S4 Fig — A. Cellulose expression was visualized by spotting 5 μl of overnight culture of S. Typhimurium, bscE mutant, E. coli MC4100, or E. coli MC4100 csgBA (LSR13) on LB supplemented with calcofluor-white and grown at 28°C for 72 hours. Colonies were visualized using a transilluminator. B. Ifnβ was quantified after stimulation of 1x10^6^ wild-type macrophages with purified curli-DNA complexes purified from S. Typhimurium, E. coli MC4100, or S. Typhimurium bscE mutant for 3 hours. Bars represent means ± S.E.M. from at least three independent experiments, * p <0.05 as determined by Students t-test. (TIFF) [file ppat.1006315.s004.tiff]
